# Methods to assess antibacterial, antifungal and antiviral surfaces in relation to touch and droplet transfer: a review, gap-analysis and suggested approaches

**DOI:** 10.1099/acmi.0.000804.v3

**Published:** 2024-07-05

**Authors:** Alexander J. Cunliffe, Peter Askew, Gillian Iredale, Abby Marchant, James Redfern

**Affiliations:** 1 Department of Natural Sciences, Manchester Metropolitan University, Manchester, M1 5GD, UK; 2 IMSL, Pale Lane, Hartley Whitney, Hants RG27 8DH, UK

**Keywords:** antimicrobial materials, antimicrobial surfaces, gap analysis, standardised test methods

## Abstract

To help assess whether a potentially antimicrobial material, surface, or coating provides antimicrobial efficacy, a number of standardised test methods have been developed internationally. Ideally, these methods should generate data that supports the materials efficacy when deployed in the intended end-use application. These methods can be categorised based on their methodological approach such as suspension tests, agar plate/zone diffusion tests, surface inoculation tests, surface growth tests or surface adhesion tests. To support those interested in antimicrobial coating efficacy, this review brings together an exhaustive list of methods (for porous and non-porous materials), exploring the methodological and environmental parameters used to quantify antibacterial, antifungal, or antiviral activity. This analysis demonstrates that antimicrobial efficacy methods that test either fungi or viruses are generally lacking, whilst methods that test bacteria, fungi and viruses are not designed to simulate end-use/lack realistic conditions. As such, a number of applications for antimicrobial activity across medical touch screens, medical textiles and gloves and transport seat textiles are explored as example applications, providing guidance on modifications to existing methods that may better simulate the intended end-use of antimicrobial materials.

## Data Summary

No new data has been reported within this article.

## Introduction

The use of antimicrobial surfaces and coatings to prevent the transfer (and potential subsequent infection [1]) of microorganisms is broad and traverses numerous applications across the built environment [1], hospitals [2], public transport [3] and high-touch devices such as mobile phones [4]. To ensure that those involved in either the production, procurement, regulation or end-use of these materials can make appropriate, evidence-based decisions, it is essential that antimicrobial surfaces and coatings are assessed using methodology that provides robust, reproducible data that reflects the efficacy intended in use [5].

To assess whether a potential antimicrobial material, surface or coating provides antimicrobial efficacy, whether that be via biocide-release, contact activity, or reduced-adhesion [6], a number of standardised test methods (STMs) have been developed internationally. Ideally, these methods should generate data that supports the materials efficacy when deployed in the intended end-use application. At the very least, they should produce reproducible data that can enable efficacy performance comparisons when these data are generated in different laboratories (e.g. undertaking a ring trial).

There is a range of different methodological approaches for testing antimicrobial efficacy described in the literature, usually framed on specific materials (e.g. ceramics [7], plastics [8], carpet fibres [9]) or antimicrobial action (e.g. silver-ion release [10], copper [11], UV irradiation [12]). In almost every instance the focus has been on optimising the function of the antimicrobial effect under laboratory/controlled conditions, rather than simulating the environment and pattern of use that might prevail when that material is placed into service. Such an approach is useful during initial research, whether that be into the active substance/mechanism or during studies on the compatibility with a final product or durability assessment. However, understanding the impact of end-use environmental conditions is important, and using a method that does not account for this may lead to inherent bias in data [13], as the antimicrobial activity demonstrated in the laboratory may well fail to be realised in practice [14]. The analysis described in this review aims to highlight the gap between existing standard test methods for assessing antimicrobial efficacy of a material and model end-use environments and suggest potential areas for method development using a number of case study applications.

## Method

A search was undertaken to collate all existing standards and established test methods relating to antimicrobial material efficacy testing by accessing standards repositories at BSOL (British Standards Online), AATCC (American Association for Textile Chemists and Colourists) and ASTM (American Society for Testing Materials). In select cases, other standards were added if they were deemed to be relevant (e.g. Japanese Industrial Standards [JIS] and ENV/JM/MONO(2007)17). Additionally, appropriate guidance documents from OECD were also included. A standard was excluded if it was assessing only the growth of microorganisms on the surface.

All relevant standards were sorted by whether they were assessing porous or non-porous surfaces (ceramics were deemed to be non-porous). They were then sorted by which category of STM was most relevant according to pre-determined definitions (Table 1). Various data (substrate type, temperature, relative humidity, incubation period and organisms used) were then extracted for each standard.

**Table 1.** Descriptions that can be used to categorise standardised methods based on the intended effect of an antimicrobial action or the end-use of the treated material

Category no. and name	Description
Category I – suspension tests	The material to be tested is immersed in a liquid containing the test species. The objective is to observe a reduction in the size of the population in the suspension.
Category II – agar plate / zone diffusion tests	The test material is placed into contact with a semi-solid growth medium that has been inoculated with the test species. The objective is to observe an effect on the growth of the organisms on the solid media (or on the test specimen).
Category III – surface inoculation tests	The test species is suspended in a liquid and then placed onto the test material. The objective is to observe a reduction in the size of the population recovered from the treated samples (often compared with no, or a smaller, reduction on the untreated ones).
Category IV – surface growth tests	The material to be tested is inoculated with a population of relevance to the material / application (either as single species or as a consortium). The inoculated samples are then incubated under conditions that encourage the growth of the organisms on the surface (either in growth chambers, flow cells or biofilm reactors). The objective is to observe the inhibition of growth on the treated sample when compared with the growth on untreated ones.
Category V – surface adhesion tests	The material to be tested is inoculated with a population of relevance to the material / application (either as single species or as a consortium). The samples are then incubated and processed to examine whether the treatment has an effect on the adhesion of the organisms to the surface (e.g. by direct microscopic examination, atomic force microscopy, etc.).

Three example end-use cases were formed based on some of the most likely scenarios for the implementation of antimicrobial materials. Whilst there are many other examples that could be described, those included in this document present a range of material types (porous and non-porous), a range of criticality (hospital wards through to mass-transport) and different contamination events (droplet transmission, direct touch transfer). In each case, an example of current practices based on the Organisation for Economic Co-operation and Development’s (OECD [15]) guidance are compared to environmental conditions and methodological decisions that would be considered more realistic in an average setting for the end-use case.

## Results and discussion

### Overview of existing standards – porous surfaces

Twenty two standards relating to porous surfaces were identified (Table 2), four of which were category one, five were category two and thirteen were category three. There were no methods that were in category four or five. The majority of the standards tested against bacteria (*n*=15), with some testing against fungi (*n*=7) and one method to test viruses. In one method both bacteria and fungi are tested, and all were using some variety of fabric or textile. Eleven standards used incubation temperature values between 35–37  °C, six standards specify a temperature of 27–30 °C and the remaining five standards specify a temperature of 20–25  °C. The relative humidity was either not stated (five standards), was submerged (i.e. relative humidity was irrelevant, three standards), or was at a high relative humidity of above 90  % (humid chamber stated in eight standards, unspecified in three standards). Three standards stated a lower relative humidity of >70  %. Most standards stated an incubation period of 24–48 h (16 standards), while two standards specified greater than 48 h and four standards stated less than 24 h. Finally, *Staphylococcus aureus* was specified in all bacterial standards among others, and *Aspergillus niger* was equivalently specified in all fungal standards among other species. The viral standard specified influenza A or feline calicivirus.

**Table 2.** Overview of STMs relating to antimicrobial efficacy of porous surfaces and coatings

Organism group	Title	Category*	STM ID	Substrate	Temp. (°C)	Humidity	Incubation period	Organism
Bacterial	Determination of antibacterial activity of textile products – absorption method	III – Surface inoculation test	ISO 20743 : 2021	All textile products	37±2	Humid chamber	18–24 h	*S. aureusK. pneumoniae*
Bacterial	Determination of antibacterial activity of textile products – transfer method	III – Surface inoculation test	ISO 20743 : 2021	All textile products	37±2	Humid chamber	18–24 h	*S. aureusK. pneumoniae*
Bacterial	Textiles – determination of antibacterial activity of antibacterial finished products – printing method	III – Surface inoculation test	ISO 20743 : 2021	All textile products	20±2	70 %	1±0.1 h2±0.1 h3±0.1 h4±0.1 h	*S. aureusK. pneumoniae*
Bacterial	Flexible cellular polymeric materials – determination of antibacterial effectiveness	I – Suspension test	ISO 23641 : 2021	Flexible cellular polymeric antibacterial treated materials	35±1	Submerged	24±1 h	*E. coliS. aureus*
Bacterial	Testing antibacterial activity and efficacy of textile products – absorption method	III – Surface inoculation test	JIS L 1902	All textile products	37±2	Humid chamber	18–24 h	*S. aureusK. pneumoniae*
Bacterial	Testing antibacterial activity and efficacy of textile products – transfer method	III – Surface inoculation test	JIS L 1902	All textile products	37±2	70 %	18–24 h	*S. aureusK. pneumoniae*
Bacterial	Testing antibacterial activity and efficacy of textile products – printing method	III – Surface inoculation test	JIS L 1902	All textile products	20±2	70 %	1±0.1 h2±0.1 h3±0.1 h4±0.1 h	*S. aureusK. pneumoniae*
Bacterial	Testing antibacterial activity and efficacy of textile products – halo method	III – Surface inoculation test	JIS L 1902	All textile products	37±2	Not stated	24–48 h	*S. aureusK. pneumoniae*
Bacterial	Quantitative method for evaluating bactericidal activity of porous / absorbent materials	III – Surface inoculation Test	IBRG TA 22–004	Textile and porous materials	35±2	> 90 %	24±1 h	*E. coliS. aureus*
Bacterial Fungal	Standard test method for using seeded-agar for the screening assessment of antimicrobial activity in carpets	I – Suspension test	ASTM E2471	Carpet textile	30±2	Submerged	24–72 h	*S. aureusSe. marcescensA. niger*
Bacterial	Fabric properties – fabrics and polymeric surfaces with antibacterial properties – characterisation and measurement of antibacterial activity	II – Agar plate / zone diffusion test	XP G 39–010	Fabric and polymeric surfaces	37±1	Humid conditions	24 h	*S. aureusK. pneumoniae*
Bacterial	Textile fabrics – determination of antibacterial activity – agar diffusion plate test	II – Agar plate / zone diffusion test	SN 195920	Impregnated textiles	37±1	Not stated	18–24 h	*S. aureusE. coli*
Bacterial	Textile fabrics – determination of antibacterial activity – germ count method	I – Suspension test	SN 195924	Textile fabrics	27	Submerged	24 h	*S. aureusE. coli*
Bacterial	Antimicrobial Activity assessment of carpets – quantitative antibacterial activity	II – Agar plate / zone diffusion test	AATCC 174	Carpet products	37	Wet fabric in closed container	6–24 h	*S. aureusK. pneumoniae*
Bacterial	Assessment of textile materials – parallel streak method	II – Agar plate / zone diffusion test	AATCC 147	Textile fabrics	37	Not stated	18–24 h	*S. aureusK. pneumoniae*
Fungal	Textiles – determination of antifungal activity of textile products – part 2 – plate count method – absorption method	III – Surface inoculation test	ISO 13629-2 : 2014	Textiles	30±2	> 95 %	48±2 h	*A. niger*
Fungal	Textiles – determination of antifungal activity of textile products – Part 2: plate count method – transfer method	III – Surface inoculation test	ISO 13629-2 : 2014	Textiles	30±2	> 95 %	48±2 h	*A. niger*
Fungal	Textile fabrics – determination of antimycotic activity – agar diffusion plate test	II – Agar plate / zone diffusion test	SN 195921	Textile fabrics	28±1	Not stated	2–7 days	*C. albicansA. niger Cl .sphaerospermum T. mentagrophytes*
Fungal	Antimicrobial activity assessment of carpets – quantitative antifungal activity	I – Suspension test	AATCC 174	Carpet products	28	Wet fabric in closed container	7 days	*A. niger*
Fungal	Determination of antifungal activity of textile products – transfer method	III – Surface inoculation test	ISO 13629-1 : 2012	Textiles	25±2	Humidity chamber	42±2 h	*A. nigerP. citrinumCl. cladosporioidesT. mentagrophytes*
Fungal	Determination of antifungal activity of textile products – absorption method	III – Surface inoculation test	ISO 13629-1 : 2012	Textiles	25±2	Not stated	42±2 h	*A. nigerP. citrinumCl. cladosporioidesT. mentagrophytes*
Viral	Textiles – determination of antiviral activity of textile products	III – Surface inoculation test	ISO 18184 : 2019	Woven, knitted and other flat textiles	25	Closed petri dish	2 h	Influenza AFeline calicivirus

**Key to species and abbreviations used in Tables 1 and 2**

**Table:** 

*Staphylococcus aureus*	*S. aureus*	Human commensal / pathogen	Common model gram- positive bacterium
*Staphylococcus epidermidis*	*S. epidermidis*	Human commensal	
*Escherichia coli*	*E. coli*	Human commensal / pathogen	Common model Gram-negative bacterium
*Klebsiella pneumoniae*	*K. pneumoniae*	Human commensal / pathogen	Common model bacterial pathogen
*Serratia marescens*	*Se. marcescens*	Environmental organism / opportunistic pathogen	
*Pseudomonas aeruginosa*	*Ps. aeruginosa*	Environmental organism / opportunistic pathogen	Common model Gram-negative bacterium
*Enterococcus faecalis*	*En. faecalis*	Human pathogen	
*Enterococcus hirae*	*En. hirae*	Human pathogen	
*Bacillus anthracis*	*B. anthracis*	Environmental organism / human pathogen	
*Bacillus subtillis*	*B. subtillis*	Environmental organism	Common model endospore-forming bacterium
*Aspergillus niger*	*A. niger*	Environmental organism / saprophyte	Common model micro-fungus
*Candida albicans*	*C. albicans*	Human commensal / pathogen	Common model pathogenic yeas
*Cladosporium sphaerospermum*	*Cl. sphaerospermum*	Environmental organism / saprophyte	
*Cladosporium cladosporioides*	*Cl. cladosporioides*	Environmental organism / saprophyte	
*Trichophyton mentagrophytes*	*T. mentagrophytes*	Dermatophyte / human pathogen	Common model pathogenic micro-fungus
*Penicillium citrinum*	*P. citrinum*	Environmental organism / saprophyte	
*Penicillium pinophylium*	*P. pinophylium*	Environmental organism / saprophyte	

### Overview of existing standards – non-porous surfaces

Eighteen relevant standards relating to non-porous surfaces were identified (Table 3), three of which were category one and fifteen were category three. There were no methods that were in category two, four, or five. The majority of the standards tested against bacteria (*n*=14), with some testing against viruses (*n*=3) and one method to test fungi. Seven standards specified a temperature of 35–37 °C, while ten standards specify 20–25  °C and the remaining standard being unspecified. The relative humidity is not stated in seven standards and is irrelevant in two standards as the materials are submerged. Of the remaining standards, six specify a high relative humidity of above 75 %, while three standards specify between 30–70  %. Seven standards specified an incubation period of 24–48 h, with the remaining eleven standards specifying less than 24 h. Ten of the bacterial standards included *Escherichia coli*, with other standards opting for *S. aureus* or *Bacillus subtilis* (although most standards accommodate multiple bacterial species). * A. niger* was used for the fungal standard and influenza A, feline calicivirus or bacteriophage Q-beta was used for the viral standards.

**Table 3.** Overview of STMs relating to antimicrobial efficacy of non-porous surfaces and coatings

Organism group	Title	Category*	STM ID	Substrate	Temp. (°C)	Humidity	Incubation period	Organism
Bacterial	Determining the antimicrobial activity of agents under dynamic contact conditions	I – Suspension test	ASTM E2149-13a	Non-leaching treated articles	35±2	Submerged	24 h	*E. coli*
Bacterial	Standard practice for determination of antibacterial activity on ceramic surfaces	III – Surface inoculation test	ASTM E3031-20	Glazed ceramics	35±2	>75 %	24±1 h	*E. coli*
Bacterial	Standard test method for determining the activity of incorporated antimicrobial agent(s) in polymeric or hydrophobic materials	III – Surface inoculation test	ASTM E2180-18	Two-dimensional hydrophobic or polymeric surfaces	37 (optimal for the species)	>75 %	24±2 h	*S. aureusK. pneumoniaePs. aeruginosa*
Bacterial	Measurement of antibacterial activity on plastics and other non-porous surfaces	III - Surface inoculation test	ISO 22196 : 2011	Plastics	35±1	> 90 %	24±1 h	*S. aureusE. coli*
Bacterial	Measurement of antibacterial activity on plastic surfaces	III – Surface inoculation test	JIS Z 2801	Plastics	35±1	> 90 %	24±1 h	*S. aureusE. coli*
Bacterial	Standardised test method for quantitative sporicidal three-step method (TSM) to determine sporicidal efficacy of liquids, liquid sprays, and vapour or gases on contaminated carrier surfaces	I – Suspension test	ASTM E2414	Solid carriers	21±3	Submerged	30 min	*B. anthracisB. subtilis*
Bacterial	Standard test methods for determination of bactericidal efficacy on the surface of medical examination gloves	III – Surface inoculation test	ASTM D7907	Examination gloves	Not stated	Not stated	0 min5 min10 min20 min30 min	*S. aureusK. pneumoniaeEn. faecalisP . aeruginosa*
Bacterial	Surfaces with biocidal properties – method for the evaluation of basic bactericidal activity of a non-porous surface	III – Surface inoculation test	NF S90-700 : 2019	Non-porous materials	18±1–25±1	30–65 %	60 min	*S. aureusEn. hiraeE. coliPs. aeruginosa*
Bacterial	Determination of bacterial reduction rate by semiconducting photocatalytic materials under indoor lighting environment – semi-dry method	III – Surface inoculation test	ISO 22551 : 2020	Indoor-light-active photocatalytic materials	25±3	50–70 %	4 h	*S. epidermidisE. coli*
Bacterial	Test method for assessing the survival of test organisms on floor covering samples	III – Surface inoculation test	WIRA test F	Floor coverings	37	Not stated	6–24 h	*E. coli*
Bacterial	Interim method for the evaluation of bactericidal activity of hard, non-porous copper-containing surface products	III – Surface inoculation test	EPA Interim	Non-porous copper	21±2	30–40 %	1–2 h	*S. aureusPs. aeruginosa*
Bacterial	Test methods for antibacterial activity of semiconducting photocatalytic materials under indoor lighting environment	III – Surface inoculation test	ISO 17094 : 2014	Indoor-light-active photocatalytic materials	25±5	Not stated	8 h	*S. aureusE. coli*
Bacterial	Test method for antibacterialactivity of semiconducting photocatalytic materials	III – Surface inoculation test	ISO 27447 : 2019	Semiconducting photocatalytic ceramics	25±3	Not stated	8 h	*S. aureusK. pneumoniaeE. coli*
Bacterial	Quantitative determination of antibacterial activity of ceramic tile surfaces – test methods – part 2: ceramic tile surfaces with incorporated photocatalytic antibacterial agents	III – Surface inoculation test	ISO 17721-2 : 2021	Glazed or unglazed photocatalytic ceramic tile surfaces	37±1	> 75 %	0.5–8 h	*S. aureusE. coli*
Fungal	Fine ceramics (advanced ceramics, advanced technical ceramics) – test method for antifungal activity of semiconducting photocatalytic materials	III – Surface inoculation test	ISO 13125 : 2013	Semiconducting photocatalytic ceramics	25±5	Not stated	3–24 h	*A. nigerP. pinophilum*
Viral	Measurement of antiviral activity on plastics and other non-porous sSurfaces	III – Surface inoculation test	ISO 21702 : 2019	Non-porous materials	25±1	> 90 %	24 h	*Infulenza AFeline calicivirus*
Viral	Determination of antiviral activity of semiconducting photocatalytic materials – test method using bacteriophage Q-beta	III – Surface inoculation test	ISO 18061 : 2014	Semiconducting photocatalytic ceramics	25±5	Not stated	4 h	BacteriophageQ-beta
Viral	Determination of activity of semiconducting photocatalytic materials under indoor lighting environment – test method using bacteriophage Q-beta	I – Surface inoculation test	ISO 18071 : 2016	Indoor-light-active photocatalytic materials	25±5	Not stated	4 h	BacteriophageQ-beta

* Category relates to the five categories of the test method described in section ‘1.2 Standardised Testing Methods’.

### Overview of existing standards – discussion

Whilst there is a relatively large number of standardised methods available for antimicrobial coating assessment, a number of methods can be seen as ‘competitive’ as they appear to address the same material / effect. In some cases, the methods can be simply substituted for each other as they are either essentially identical (e.g. JIS Z 2801 [16] and ISO 22196 [17]) or they are capable of providing a similar amount of information with regards to the basic antimicrobial activity of a certain material. Conversely, some are very specific to a certain type of material or antimicrobial mechanism (e.g. ISO 27447 [18]). Whilst some element of global harmonisation towards method development exists, the development of STMs can operate on a regional/national basis (e.g. ASTM methods in the USA [19], NSF Norms in France [20]), the presence of certain trade organisations (e.g. AATCC in the USA [21], representing the textile and carpeting industries), or the need for pass criteria to satisfy certain brand-marks (e.g. the Kohkin brand mark associated with the ‘pass’ level in JIS Z 2801, or the criteria with [22,22] in Japan). In some cases, certain active substance producers have encouraged the development of standard methods that work well with their technology (presumably in the hope of gaining a competitive advantage in the market). A number of these regional methods have been converted into international norms (often stripped of their arbitrary pass / fail criteria) such as JIS Z 2801 ->ISO 22196, and [22] ->ISO 20743 [23] (combined with part of a French national standard). In some cases, the standards have been normalised further such as in the OECD tier one method for treated articles which presents a base method for both non-porous and porous materials through the harmonisation of the parameters used in ISO 22196 and the absorption method described in ISO 20743.

As described in Tables 2 and 3, there are numerous STMs available to examine the basic antimicrobial properties of treated materials, coatings, textiles, etc. Some can even be used to simulate actual exposure conditions (e.g. a flow cell biofilm method may be capable of accurately simulating the conditions present in pipelines or catheters). However, in most cases the methods only look at basic antimicrobial properties and do not simulate end-use scenarios sufficiently well to be capable of predicting performance in practice nor in supporting any claims made during product / material registration. Some approaches have been described that start to address this, but only in guideline form (OECD Guidance / ECHA Guidance / Nordic Council Guidance). As such, taking an application or environment and considering in detail how an antimicrobial test method may be developed, and what the environmental parameters may be is an essential step forward for those interested in antimicrobial coatings.

### Example end-use scenario one: touch screen of a heart monitor in ICU

Most touch screens are constructed from materials that are essentially non-porous [24]. In a clinical setting, they will be subject to low to moderate interaction and although human skin contact will occur, in many cases the operative will probably be wearing a disposable / surgical glove [25]. As such, whilst some skin flora may be transferred to the touch screen, most of the microbial contamination delivered by touch is likely to be transferred from other surfaces via a glove [26]. Deposition of microorganisms from the air can occur and it is possible that droplets (respiratory, etc.) could also be deposited on the touch screen [27]. Most of the transfer / deposition will either be dry or be associated with very transient wetness. The environmental conditions within an ICU are likely to be constant [28,30] and although there may be some air movement from ventilation systems, they will be of low velocity [31]. Temperature may range from 16 °C to 25 °C and relative humidity likely between 30 and 60% [32].

As described in Table 3, there is no single STM that has exposure conditions that match those anticipated from the touch screen. ISO 22196 is intended for use with non-porous materials, but the exposure conditions require the full hydration of the surface of the material, and even if the temperature and contact time were aligned to those of a hospital ICU, it would still be a poor model due to the volume of liquid applied. In contrast, NSF S90-700 uses small droplets as an inoculum and so might be suited for simulating aerosol / droplet deposition but has some arbitrary time to dryness requirements and has no ‘dry’ contact component.

**Fig. 1.**
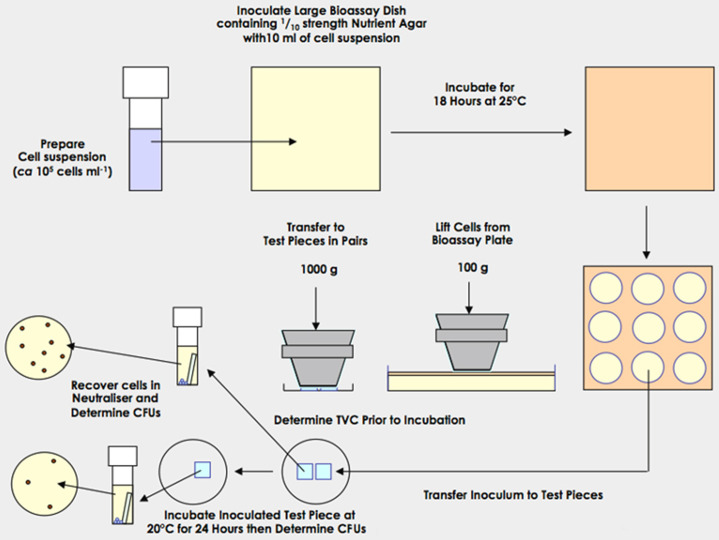
A hand contact simulation protocol designed to assess the efficacy of non-porous antimicrobial surfaces.

**Fig. 2.**
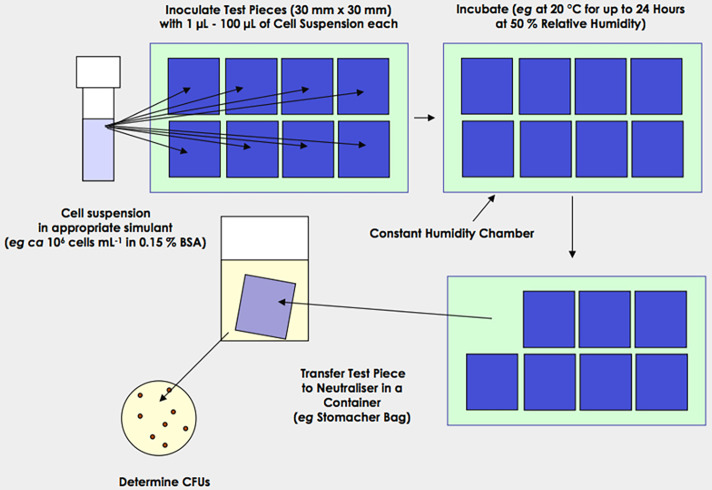
A simulated splash protocol designed to assess the efficacy of non-porous antimicrobial surfaces.

A method which simulates hand contact (Fig. 1) would likely present an extreme worst-case as a much larger number of organisms will be transferred than is likely to occur in an ICU [33]. A further modification in which an intermediate, untreated surface is employed may provide a more representative model. This surface would be contaminated by either using a splash method (e.g. Fig. 2) or by applying a wet inoculum to the surface and allowing it to dry and using the resulting deposit as the inoculum to pick up using the transfer device. However, this would require significant additional work (parameters are described in Tables 4, 5 and 6).

**Table 4.** Comparison of approaches regarding the use of antimicrobial coatings for touch-screens via droplet deposition

Parameters	OECD example	Touch-screen	Modification
Environmental	20 °C and 50 % RH	20–24 °C and 50 % RH	Use 24 °C as this will accelerate drying and so present the worst-case for a technology to function under and 50 % RH.
Volume of droplet	50 µl	Droplets likely to be 1 µl or smaller	Use 1 µl as base model and look at aerosol deposition if functionality is detected.
Species used	*Ps. aeruginosaA. baumanniiEn. hiraeSt. pneumoniae*MRSA	*Ps. aeruginosaA. baumanniiEn. hiraeSt. pneumoniae*MRSA	Use species in example.
Suspending medium	Water	Respiratory droplets	A low concentration of either albumin or mucin will be employed.
Contact times	0, 6, 12, 24 h	Maximum of 1 h	0, 5, 10, 30, 60 min will be used.
Sample form	Small coupons	Small coupons	No modification.
Recovery method	Samples ‘extracted’ in a neutralizer	Samples ‘extracted’ in a neutralizer	No modification.

**Table 5.** Contamination of touch-screens via hand deposition of bacteria

Parameters	OECD example	Touch-Screen	Modification
Environmental	20 °C and 50 % RH	20–24 °C and 50 % RH	Use 24 °C as this will accelerate drying and so present the worst-case for a technology to function under and 50 % RH.
Transfer medium	Plastic food-wrap	Latex glove	A section of latex glove will be attached to the transfer device.
Species used	MRSA	*Ps. aeruginosaA. baumanniiEn. hirae*MRSA	Use additional species. Will need to perform pre-tests to establish incubation time to establish mono-layers on bioassay plates.
Contact times	0, 15, 30, 60 min.	Maximum of 1 h.	0, 5, 10, 30, 60 min will be used.
Sample form	Small coupons	Small coupons	No modification.
Recovery method	Samples ‘extracted’ in a neutralizer	Samples ‘extracted’ in a neutralizer	No modification.

This method could also be applied to determine the antiviral activity of a surface in a similar manner to bridge a further gap in current methodologies (Table 6).

**Table 6.** Contamination of touch-screens via hand deposition of viruses

Parameters	OECD example	Touch-screen	Modification
Environmental	20 °C and 50 % RH	20–24 °C and 50 % RH	Use 24 °C as this will accelerate drying and so present the worst-case for a technology to function under 50 % RH.
Transfer medium	Plastic food-wrap	Latex glove	A section of latex glove will be attached to the transfer device.
Species used	MRSA	phi6MS2Relevant mammalian virus	Bacteriophage / virus employed in-place of bacteria. Use of an enveloped and non-enveloped bacteriophage to provide data for a broad rage of species. Additional species of concern could also be employed.
Contact times	0, 15, 30, 60 min	Maximum of 1 h	0, 5, 10, 30, 60 min will be used.
Sample form	Small coupons	Small coupons	No modification.
Recovery method	Samples ‘extracted’ in a neutralizer	Samples ‘extracted’ in a neutralizer	No modification.

### Example end-use scenario two: medical uniforms (scrubs)

Medical uniforms are commonly produced from polyester / cotton blends [34]. They are normally porous and have a high moisture holding capacity. Over the course of a shift (8–12 h) it is likely that a medical professional would come into close contact with tens if not hundreds of patients, visitors, and colleagues where the potential for a contaminating event is high [35]. Additionally, medical uniforms exist between two distinct environments (i) that of the wearer (body temperature, sweat/humidity) and (ii) the physical environment within which the wearer is placed (with different temperatures, humidity, etc.). All these factors are going to change how contaminating microorganisms interact with an antimicrobial textile, which should be considered when thinking about the efficacy assessment of antimicrobial activity [36]. As the wearer is only likely to be wearing the uniform for one shift at a time, for example between 8–12 h, antimicrobial activity that takes longer than this may not be beneficial. A droplet deposition method would be well suited to simulate a contamination event rather than a fully wet test. If a gross contamination event occurred resulting in a wet uniform for a prolonged period, it is likely that the garment would be laundered [37]. An antimicrobial effect may still be advantageous under these fully wet conditions to reduce the risk to the laundry staff who are involved in the laundering process. This activity could be determined by employing methods such as: IBRG TA22-004 [38], ISO 20743 [23], AATCC 100 [22,22, 39] and the OECD method (ENV/JM/MONO (2014)18 [15]). However, in each case the method does not provide realistic environmental conditions, and as such, modifications may be required (Tables 7, 8 and 9).

**Table 7.** Contamination of medical uniforms by blood

Parameters	ISO 22196	Medical uniforms	Modification
Environmental	35 °C and >90 % RH	20–24 °C and 50 % RH	Use 24 °C as this will accelerate drying and so present the worst-case for a technology to function under and 50 % RH.
Volume of droplet	400 µl	400 µl	No modification.
Species used	*S. aureusE coli*	*S. aureusE. coliPs. aeruginosaEn. hiraeA. baumannii*	Use additional species relevant to the medical field.
Suspending medium	1/500 NB	Blood	A more suitable suspending medium will be employed to more closely simulate the contamination event.
Contact times	24 h	24 h	No modification.
Sample form	Small coupons	Small coupons	No modification.
Recovery method	Samples ‘extracted’ in a neutralizer	Samples ‘extracted’ in a neutralizer	No modification.

**Table 8.** Gap analysis for medical uniforms contaminated with droplets

Parameters	OECD example	Medical uniform	Modification
Environmental	20 °C and 50 % RH	20–24 °C and 50 % RH	Use 24 °C as this will accelerate drying and so present the worst-case for a technology to function under and 50 % RH.
Volume of droplet	50 µl	Droplets likely to be 1 µl or smaller	Use 1 µl as base model and look at aerosol deposition if functionality is detected.
Species used	*Ps. aeruginosaA. baumanniiEn. hiraeSt. pneumoniae*MRSA	*Ps. aeruginosaA. baumanniiEn. hiraeSt. pneumoniae*MRSA	Use species in example, additional species of interested could also be tested (phi6 and MS2 for example to explore viral activity).
Suspending medium	Water	Respiratory droplets	A low concentration of either albumin or mucin will be employed.
Contact times	0, 6, 12, 24 h	Maximum of 8 h	0, 0.5, 1, 2, 4, 8 h will be used.
Sample form	Small coupons	Small coupons	No modification.
Recovery method	Samples ‘extracted’ in a neutralizer	Samples ‘extracted’ in a neutralizer	No modification.

**Table 9.** Gap analysis for medical uniforms contaminated via surface to surface interaction

Parameters	ISO 20743 (Printing Method)	Medical uniform	Modification
Environmental	20±2 °C70 % RH	20–24 °C and 50 % RH	Use 24 °C at 50 % RH.
Transfer medium	Filter paper	Textile or representative non-porous surface	Inoculation surface chosen based on interaction under investigation, e.g. bedding – uniform or non-porous fomite – uniform.
Species used	*S. aureusK. pneumoniae*	*S. aureusK. pneumoniae*	Use species in example, additional species of interested could also be tested (phi6 and MS2 for example to explore viral activity).
Contact times	1±0.1, 2±0.1, 3±0.1 or 4±0.1 h	Maximum of 8 h	0, 0.5, 1, 2, 4 and 8 h will be used.
Sample form	Small coupons	Small coupons	No modification.
Recovery method	Samples ‘extracted’ in a neutralizer	Samples ‘extracted’ in a neutralizer	No modification.

Using a common method such as ISO 20743 would enable reproducible efficacy assessment, but as described above, the conditions the method requires would not be comparable to a uniform in a medical setting. In this scenario, various modifications can be suggested (Table 7). Target species can be selected to better simulate those considered important; nosocomial pathogens [40], suspended in more complex media (e.g. artificial blood [41], urine [42] etc.). Additionally, the temperature can be lowered to be more realistic of a ‘warm day’ which will accelerate drying onto the material [43]. Assessment of the antimicrobial activity of fabric following ageing [44] will also need to be determined to ensure activity throughout the lifetime of the garment is achieved. Current standards such as AATCC 61 [45], which is a method that employs accelerated laundering to determine the durability of a textile that is expected to undergo frequent laundering, could be employed to age the textile prior to efficacy studies.

Like the droplet deposition method described for touch screen applications in the medical setting, the OECD method can be used to consider the same droplet contaminating event on a medical uniform (Table 8). Like the modifications described in Table 7, selecting a temperature to accelerate drying of droplets and selecting a more complex suspending medium may be suitable. Additionally, reducing the volume of inoculum may better simulate aerosol deposition of droplets [46]. Due to the intended use-time of a medical uniform, contact time can be reduced to a maximum of 8 h.

The nature of the environment means that those wearing medical uniforms will inevitably contact surfaces that are contaminated with pathogens. For example, this may occur when a medical professional is providing care to a patient in a bed, where the uniform may come in direct contact with both the bed linen (textile [47]) and bed rails (non-porous materials [48]). In either of these scenarios, a medical uniform that can actively kill contaminating microorganisms would be beneficial. However, despite both contamination events potentially co-occurring within a physically close space and time, the transfer of microorganisms from textile-to-textile and non-porous material-to-textile is different, and as such, efficacy assessment should consider these scenarios within the methodology (Table 9). The ISO 20743 standard defines three test methods, the absorption method is a fully wet method, the transfer method removes cells from an agar plate and transfers them to the test surface in a similar manner to the OECD hand contact simulation method, and a printing method where the bacterial cells are placed on a filter paper and then transferred onto the test fabric by printing using a defined weight. This method has the least moisture and nutrients transferred along with the inoculum.

### Example end-use scenario three: woven seat cover in a train

Most seat covers used on public transport are constructed from materials that are essentially porous woven fabrics although some may be coated causing them to act more like non-porous surfaces [49]. In a public setting, porous seat covers will be subjected to periods of high use and moisture, for example soiling from food and drink spillages or passengers wearing wet clothing [50]. However, there will be periods of the day when usage will be low (e.g. overnight [51]). Most of the microbial contamination will probably be delivered by touch, the contamination will likely be transferred from porous and non-porous surfaces such as trousers, coats and other clothing, hand contact, settling from the air as well as droplets from coughs and sneezes [52]. Most of the transfer / deposition will either be dry contamination or will be associated with very transient wetness, although it is possible that fully wet events may occur on occasion. The environmental conditions in public transport scenarios are likely to be reasonably constant with continual air movement during periods of use. Temperature will probably be between 20 °C and 30 °C and ambient humidity will probably be around 50 % relative humidity although these factors would change depending on region and season [53].

It can be seen from Table 2 that no single STM has exposure conditions that match those described above to simulate the exposure scenarios described for porous seat covers. IBRG TA22-004 and ISO 20743 as well as AATCC 100, JIS L 1902, and the OECD method (ENV/JM/MONO (2014)18) are intended for use with porous materials, but the exposure conditions require the full hydration of the surface of the material at high relative humidities at relatively high temperatures for prolonged periods of time and, even if the temperature and contact time were modified, it would still be a poor model due the volume of liquid applied and lack of a drying effect simulation which would naturally occur in reality.

End-use simulation methods described in the OECD / ECHA guidance documents with modification could be used to determine the antibacterial activity of woven fabrics that become contaminated with either small splashes of contaminated liquid or by hand contact (Table 10). Such approaches would seem to provide a more suitable starting point from which to build an appropriate testing model, ensuring activity over time is assessed to understand the function of the technology better. A reduction in the size of the contaminating microorganisms during usage period would be optimal but reductions during periods of non-use would also be beneficial.

**Table 10.** Gap analysis for woven seat covers via droplet deposition

Parameters	OECD example	Woven seat cover	Modification
Environmental.	20 °C and 50 % RH	20–30 °C and 50–70 % RH	Use 25 °C as this will accelerate drying and so present the worst-case for a technology to function under and 50 % RH.
Volume of droplet	50 µl	Droplets likely to be 1 µl or smaller	Use 1 µl as base model and look at aerosol deposition if functionality is detected.
Species used	*Ps. aeruginosaA. baumanniiEn. hiraeSt. pneumoniae*MRSA	*S. aureusE. coliPs. aeruginosaEn. hirae*	Species selected for their relevance to the environment and as representative species for Gram-negative and Gram-positive species (EN 1276 disinfection species), additional species of interested could also be tested (phi6 and MS2 for example to explore viral activity).
Suspending medium	Water	Respiratory droplets	A low concentration of either albumin or mucin will be employed.
Contact times	0, 6, 12, 24 h	Maximum of 8 h	0, 0.5, 1, 2, 4 and 8 h will be used.
Sample form	Small coupons	Small coupons	No modification.
Recovery method	Samples ‘extracted’ in a neutralizer	Samples ‘extracted’ in a neutralizer	No modification.

### Observations and challenges regarding antifungal methods

With regards to testing fungicidal activity on surfaces (both porous and non-porous), it can be seen from Table 2 that few tests exist that determine activity against fungal isolates (including fungal spores). The majority of STMs in Table 2 that measure activity against fungi describe methods to determine the efficacy of treated surfaces to prevent fungal growth rather than the efficacy of treated surfaces to reduce the viability of fungal spores. Although preventing growth on the surface of the substrate is an obvious benefit, which prevents the degradation of the material itself and prevents large reservoirs of organisms developing over time, exhibiting an effect against the fungi only once a spore has germinated would not remove the risk of cross contamination. It may be possible to modify existing antibacterial standard methods so that sporicidal action could be investigated.

## Conclusion

These example applications are not intended to be exhaustive but act as a guide during the early stages of development of new antimicrobial materials and coatings. It is clear from the examples described that when deciding which efficacy test to perform, it is important to have a clearly defined set of environmental and exposure parameters in which the surface needs to perform and from which antimicrobial claims will be made, ideally designed / modified to simulate as closely as possible the desired exposure scenario. However, most existing methods and approaches are lacking in this respect.

Often surface coatings will be developed with the intention of providing a benefit in a wide range of environments that could be contaminated in a variety of ways in each of these environments. It would be impractical and unreasonable to expect the producer to perform tests to determine the efficacy under every eventuality. It is therefore necessary in cases where a broad claim will be made to identify the worst-case and to determine which tests should be employed under these scenarios.

This review and meta-analysis demonstrates that whilst significant efforts to develop standardized antimicrobial test methods have been made, decisions regarding which existing methodology to use may need to consider modification to match the requirements of the new materials and provides guidance for the activities required to develop new, and more appropriate, testing methodologies.

## Abbreviations

AATCC: American Association for Textile Chemists and Colourists
ASTM: American Society for Testing Materials
ECHA: European Chemicals Agency
IBRG: International Biodeterioration Research Group
ISO: International Organisation for Standardization
JIS: Japanese Industrial Standards
OECD: Organisation for Economic Co-operation and Development
RH: relative humidity
STM: standardised test method
